# Next Generation Sequencing for the Prediction of the Antibiotic Resistance in *Helicobacter pylori*: A Literature Review

**DOI:** 10.3390/antibiotics10040437

**Published:** 2021-04-14

**Authors:** Ilaria Maria Saracino, Matteo Pavoni, Angelo Zullo, Giulia Fiorini, Tiziana Lazzarotto, Claudio Borghi, Dino Vaira

**Affiliations:** 1 Microbiology Unit, Department of Specialized, Experimental, and Diagnostic Medicine, IRCCS St. Orsola Polyclinic, University of Bologna, 40138 Bologna, Italy; ilariamaria.saracino@studio.unibo.it (I.M.S.); tiziana.lazzarotto@unibo.it (T.L.); 2 Department of Medical and Surgical Sciences, IRCCS St. Orsola Polyclinic, University of Bologna, 40138 Bologna, Italy; matteo.pavoni@studio.unibo.it (M.P.); giulia.fiorini@aosp.bo.it (G.F.); claudio.borghi@unibo.it (C.B.); 3 Gastroenterology and Digestive Endoscopy, ‘Nuovo Regina Margherita’ Hospital, 00153 Rome, Italy; angelomario.zullo@aslroma1.it

**Keywords:** *H. pylori* antibiotic-resistance, molecular methods, nxt generation sequencing, whole genome sequencing

## Abstract

**Background and aims**: Only a few antimicrobials are effective against *H. pylori*, and antibiotic resistance is an increasing problem for eradication therapies. In 2017, the World Health Organization categorized clarithromycin resistant *H. pylori* as a “high-priority” bacterium. Standard antimicrobial susceptibility testing can be used to prescribe appropriate therapies but is currently recommended only after the second therapeutic failure. *H. pylori* is, in fact, a “fastidious” microorganism; culture methods are time-consuming and technically challenging. The advent of molecular biology techniques has enabled the identification of molecular mechanisms underlying the observed phenotypic resistance to antibiotics in *H. pylori*. The aim of this literature review is to summarize the results of original articles published in the last ten years, regarding the use of Next Generation Sequencing, in particular of the whole genome, to predict the antibiotic resistance in *H. pylori.* **Methods**: a literature research was made on PubMed. The research was focused on II and III generation sequencing of the whole *H. pylori* genome. **Results**: Next Generation Sequencing enabled the detection of novel, rare and complex resistance mechanisms. The prediction of resistance to clarithromycin, levofloxacin and amoxicillin is accurate; for other antimicrobials, such as metronidazole, rifabutin and tetracycline, potential genetic determinants of the resistant status need further investigation.

## 1. Introduction

Antibiotic resistance is an increasing problem for *H. pylori* eradication therapies. Standard antimicrobial susceptibility testing (AST) can be used to prescribe appropriate therapies but is currently recommended only after the second therapeutic failure [1] because it requires an invasive test such as endoscopy, and culture methods are challenging and time-consuming [2]. The advent of molecular biology techniques has enabled the identification of molecular mechanisms underlying the observed phenotypic resistance to antibiotics in *H. pylori.* Aim of this literature review is to summarize the results of original articles published in the last ten years, using the whole genome Next Generation Sequencing (NGS) to predict antibiotic resistance in *H. pylori*.

## 2. Results

### 2.1. Clarithromycin

Clarithromycin (CLA) is a bacteriostatic antibiotic, it belongs to the group of macrolides that bind reversibly to the peptidyl transferase loop of domain V of the 23SrRNA, interfering with protein elongation and blocking protein synthesis. Compared with other macrolides, clarithromycin is better absorbed in the gastric mucus layer and is more acid-stable [3]. Investigating clarithromycin resistance with traditional molecular methods, a high number of mutations have been detected in the macrolide-binding site of 23SrRNA gene, i.e., A2142G, A2142C, A2143G, T1942C, G1939A, C2147G, G2172T, T2182C, A2116G, A2144G/T, A2115G, G2111A, T2717C, T2289C, G2224A, and C2245T (in accordance to Taylor et al. numbering) [4,5,6]. In general, NGS has confirmed that clarithromycin resistance is mainly based on point mutations in nucleotide positions 2142 (A to G/C) and 2143 (A to G) in the 23SrRNA gene [7,8,9]. The commercially available qPCR (quantitative Polymerase Chain Reaction) assays for the detection of A2142C/G and A2143G, would be sufficient to monitor CLA resistance in clinical practice; but only a limited number of nucleotide position can be analysed with this tool, while whole genome sequencing (WGS) delivers a more comprehensive description of resistance determinants present in a clinical isolate and may detect new mutations potentially conferring drug resistance. In addition, the clinical relevance of new mutations can easily be assessed by retrospective analysis of WGS data [10]. In fact, mutations of insertion or deletion in *rpl22* gene (encoding a ribosomal protein that interacts with the 23SrRNA domains) and a “G to A”point mutation in *infB* gene (encoding a translation initiation factor, IF-2) were detected with WGS in some CLA-resistant *H. pylori* strains carrying a wild type 23SrRNA [11]. On the other hand, it has been reported that five conserved families of multidrug efflux pump transporters can contribute to antibiotic resistance in *H. pylori*. One of these, the resistance-nodulation-cell division (RND) family, forms a homotrimer with an outer membrane protein (TolC) and a periplasmic membrane fusion protein (AcrA) [12,13,14]. Currently, using WGS, four-gene clusters of efflux pump systems (hp0605-hp0607, hp0971-hp0969, hp1327-hp1329, and hp1489-hp1487) [15,16] have been identified as belonging to the RND family in *H. pylori*, and linked to the development of resistance to CLA [17]. With WGS analysis, Chen et al. (2018) [15] failed to find significant differences in gene mutations of RND family between CLA-susceptible and CLA-resistant phenotype. However, the number of mutations in the RND family was significantly higher in *H. pylori* strains carrying the A2143G point mutation of the 23SrRNA gene. Moreover, Iwamoto et al. (2014) [12], using the same type of approach, observed a significant difference in the number of SNVs (single-nucleotide variant) of the hp0605-hp0607 cluster between susceptible and resistant *H. pylori* strains.

### 2.2. Metronidazole

Metronidazole (MZ) and tinidazole are bactericidal chemotherapic compounds that belong to the nitroimidazole group of drugs, their antimicrobial activity is only marginally affected by low pH. Nitroimidazoles are prodrugs that need to be activated within the target cell by one or two electron transfer processes. This reduction leads to the formation of nitro-anion radicals and imidazole intermediates that cause lethal damage to subcellular structures and DNA [18,19,20]. The prediction of metronidazole resistance based on genotypic information still remains challenging. Various mutational changes affecting several genes have been shown or hypothesized as being involved in the development of MZ resistance in *H. pylori* [5,21,22]. Traditional molecular techniques identified *rdxa* (oxygen-insensitive NAD(P)H nitroreductase) and *frxa* (NAD(P)H flavin nitroreductase) genes [23] as putative MZ-resistance determinants. Whole genome NGS enabled the detection of complex variants in *gyrA* and *frxA* genes, as well as the study of mutations in other genes, i.e., *recA* (RecA protein for the repair and maintenance of DNA), *sodB* (superoxide dismutase B), *fur* (ferric uptake regulator protein), *mdaB* (NADPH:quinone oxidoreductase MdaB), *ribF* (Riboflavin biosynthesis protein), *omp11* (Outer membrane protein 11), and *rpsU* (30S ribosomal protein S21). All genes hypothetically related to MZ resistance encode putative MZ-reducing enzymes, DNA repair proteins, proteins regulating cell responses to oxidative stress, and outer membrane proteins [24]. Most metronidazole-resistant *H. pylori* strains carry multiple *rdxA* and *frxA* mutations. In particular, frameshift mutations (i.e., at codon positions 105, 149 or 192 in *frxA* and 18, 38 and 112 in *rdxA*) and point mutations resulting in amino acid exchanges (i.e., A67V, A68E, K64N, P106S, R90S and R16C/H in the *rdxA* gene) were observed only in metronidazole resistant strains [25,26]. Other mutations (i.e., at codon positions 18 in *frxA* and 62, 96 and 162 in *rdxA*) were distributed between resistant and susceptible strains [5,7,25]. Null mutations in *rdxa* functional sites (frameshift, premature stop, large deletion, large sequence insertions ending with a stop and point-mutations) leading to a loss of binding sites for FMN (flavin mononucleotide) are predictive of MZ-resistance. The same types of mutations were detected also in the *frxa* gene, but with lower predictivity for phenotypic resistance. The role of the other putative MZ-resistance genes is even less evident [27]. Metronidazole-resistant strains without mutations in *rdxA* and/or *frxA* were also reported, and downregulation of *rdxA* expression or mutations in other genes has been suggested [12,27,28,29]. Chua et al. (2019) [27], in fact, identified through whole genome sequencing four protein clusters harbouring a variable site in which the distribution of amino acid variants was significantly greater among the MZ-R strains: D85N in the inner-membrane protein RclC, V265I in a biotin carboxylase protein and A51V/T in HP0918 (hypothetical protein involved in tRNA biosynthesis). Overall, these observations suggest that MZ resistance is multifaceted and more studies are needed to investigate the association between gene polymorphisms and metronidazole resistance.

### 2.3. Levofloxacin

Levofloxacin (LEVO) is a broad-spectrum antibiotic which belongs to the third generation of the quinolones group, namely fluoroquinolones. They are bactericidal antibiotics that exert their antimicrobial activity by inhibition of type II topoisomerases (DNA gyrase and topoisomerase IV). *H. pylori* lacks topoisomerase IV, so fluoroquinolone resistance is likely due to mutations in the DNA gyrase. This enzyme is a tetramer that consists of two A subunits and two B subunits, encoded by the *gyrA* and *gyrB* genes. The main function of this enzyme is to catalyse the negative supercoiling of DNA [30]. Using conventional molecular methods, point mutations in the regions of *gyrA* gene encoding amino acids 87, 88, 91 and 97 had been proposed as putative levofloxacin-resistance determinants. In *gyrA* and *gyrB*, quinolone resistance-determining regions (QRDR) were identified. *GyrA* QRDR goes from codons A71 to Q110; *gyrB* QRDR goes from codons E415 to S454. With NGS techniques, mutations inside and outside the QRDR of both *gyrA* and *gyrB* genes were analysed and associated with levofloxacin resistance in *H. pylori* [5]. Amino acid exchanges at codon 87 and/or 91 (i.e., N87I/N87K/D91Y/D91N/D91G) in the QRDR of the *gyrA* gene were confirmed as determinants of the resistant status [5,25]. Strains with mutations in both positions generally show higher levels of resistance. Qumar et al. (2020) [31], using whole genome NGS, also identified a N-terminal extension of GyrA by five amino acid residues (QDNSV) that occurred solely in LEVO-R *H. pylori* strains. This insertion caused a conformational change in the GyrA protein, reducing its binding affinity to fluoroquinolone antibiotics. Mutations in the QRDR of *gyrB* gene (i.e., D435N and V437L), and mutations outside of it (i.e., R484K and R579C), are not so strictly correlated to the phenotypic resistance; and it appears that their presence has no synergistic effect with *gyrA* mutations [32]. On the other hand, it was suggested that genotypic and phenotypic LEVO-resistance statuses are highly concordant only when considering mutations of the QRDR sequence in both *gyrA* and *gyrB*. Moreover, a small portion of phenotypically resistant strains carrying wild type *gyrA* and *gyrB* genes were reported, suggesting the involvement of other mechanisms in the onset of LEVO-resistance [5]. In many Gram-negative bacteria, the presence of efflux pumps has been reported to confer the fluoroquinolone resistance [33], but this mechanism seems to be rather unlikely in *H. pylori* [16,34]. On the other hand, resistance to quinolone drugs may be attributed to the change in membrane permeability, which may also play a role in multi-drug resistance (shown by a decrease in drug accumulation inside the cells) and alteration of the outer membrane protein (OMP) [35,36,37]. Overall, genotype analysis based on QRDRs of *gyrA* and *gyrB* genes gives a high predictivity of the LEVO-R status in *H. pylori* [5].

### 2.4. Amoxicillin

Amoxicillin (AM) is a bactericidal antibiotic that belongs to the penicillin group of drugs. The drug binds to penicillin binding proteins (PBPs) and interferes with bacterial cell wall synthesis, resulting in lysis of replicating bacteria. The antibacterial activity of amoxicillin is much the same as that of other penicillins, but amoxicillin is better released in the gastric juice, and displays increased stability in acidic conditions compared with other penicillins [38]. In *H. pylori*, there is no evidence that amoxicillin resistance is caused by beta-lactamase activity [39]. Many *H. pylori* isolates described as being amoxicillin resistant are often only tolerant to penicillins due the absence of PBP 4 [40]. Stable amoxicillin resistance in *H. pylori* is rare and seems to be mainly mediated by alterations to PBPs. Mutations in (or adjacent to), three motifs “SXN/SXXK/KTG” in PBP1A, PBP2 and PBP3 were identified as putative AM-resistance conferring determinants, together with mechanisms of reduced membrane permeability [36,41,42]. By using WGS, it was confirmed that structural alterations within the PBP1A were relevant for AMX-R, in particular in those belonging to three PBP-motifs (SAIK368_371, SKN402_404, KTG555_557, and SNN559_561) and at C-terminus codons (A474, T558, T593, and G595). Moreover, new putative AM-R genotypes were detected, i.e., F366L, S405N, A474T, T558S and N562H [5,43]. Tshibangu-Kabamba et al. (2020) [5] using WGS, also observed mutations at five codons (S402G, S414R, T556S, N562Y, and T593A/G/K/S) that were previously linked to AM-resistance by natural transformation. Saranathan et al. (2020) [7] detected, in AM-resistant strains, point mutations in genes *pbp1* (N107R, A201V, V250I, S543T), *pbp2* (I259T), and/or *pbp3* (D2N, A50S, F490Y, A541T, V374I) located near the AM-resistance motifs. With WGS, also other mechanisms were observed inducing in vitro high-level AM-resistance, they included mutations in *hofH* (outer membrane protein), *hefC* (RND pump protein) and *hopC* (outer membrane protein) genes. Overall, the analysis of *pbp1A*, *hofH*, *hefC* and *hopC* genes gives a good predictivity of the AM-resistant phenotype [44].

### 2.5. Rifabutin

Rifabutin (RIFA) belongs to the “rifamycin-group” of bactericidal antibiotics (together with rifamycin, rifampicin, and rifaximin) that bind the β-subunit of the DNA-dependent RNA polymerase, leading to the inhibition of the transcription. Rifabutin is a recommended drug for *H. pylori* rescue therapies in regions with high quinolone resistance rates, because its antibacterial activity is not affected by the low pH and is higher than that of rifampicin [1]. The β-subunit of the DNA-dependent RNA polymerase complex is encoded by the *rpoB* gene [45]. Although increasing, the resistance against rifamycins is very rare [46]. In *H. pylori*, resistance to rifampicin has been associated with amino acid exchanges in the rifampicin resistance determining region (RRDR) of the *rpoB* gene [25], mainly at codons 525 to 545, 547and 586. Lauener et al. (2018) [25], through WGS, observed that amino acid substitutions L525P and H540N were associated with rifampicin resistance (MIC >32 mg/L). By considering a 4 mg/L clinical breakpoint (instead of 1 mg/L, as established by EUCAST) [47], it was possible to find an agreement between the WGS results and phenotypic rifampicin susceptibility testing. Cross-resistance between rifabutin and rifampicin was frequently reported [48,49]. With NGS, Miftahussurur et al. (2019) [50] detected several mutations in *rpoB* gene associated with rifamycin resistance (i.e., I2619V, V2592L, T2537A, F2538L, K2359S6, K2594R7, D2381E8, T1540A, N2603D and E2809D) that were located outside the known RRDR and were not related to high-level resistance (MIC ≥ 16 mg/L). It was suggested that some of these mutations (i.e., V2592L, T2537A, and F2538 L) might be related to rifaximin, but not rifabutin resistance. In another study, using the same type of approach, these authors identified point mutations (i.e., I837V, A2414T, K2068R, Q2079K) leading to rifaximin—but not rifabutin—resistance, suggesting the lack of cross-resistance between rifaximin and rifabutin [51].

### 2.6. Tetracycline

Tetracycline (TC), a bacteriostatic antibiotic that binds to the 16SrRNA, inhibiting protein synthesis, has been intensively used since the 1950s, and many bacterial pathogens have acquired resistance to this antibiotic [52]. In most species, tetracycline resistance is obtained in two main modes of action: efflux systems, or ribosomal protection proteins [53]. In the case of *H. pylori*, TC resistance is not observed as frequently as in other bacteria, although its prevalence is slowly increasing [54]. In *H. pylor*i, resistance to TC seems to be conferred by mutations in the 16rRNA gene, in particular a triple mutation AGA965 to 967TTC (h31 loop) and a G942 deletion (*E. coli* numbering) both located in domain III, and several nucleotides in this region interact with tRNA molecules [55]. Genotype-based prediction of TC resistance is difficult, also with NGS, this is due to the low resistance rate to this antibiotic, moreover isolates without mutations at these nucleotide positions in the 16SrRNA gene display a resistant phenotype [7,56]. Therefore, tetracycline resistance seems to be multifactorial, involving alterations in ribosomal binding, enzymatic degradation of antibiotics, a reduction of membrane permeability, and an active efflux, and need further investigation [25,57,58].

## 3. Discussion

*H. pylori* infection is correlated to upper gastrointestinal diseases such as gastritis, peptic ulcers, gastric MALT (mucosa-associated lymphoid tissue) lymphoma, gastric cancer [59] and other extra-gastric complications [60]. In 2020, the CDC stated that around two-thirds of the World’s population are actually infected [61]. In 2017, the World Health Organization categorized clarithromycin resistant *H. pylori* as a “high-priority” bacterium [62]. Antibiotic resistance (AR) is an increasing problem for eradication therapies, and the trending abuse of antibiotics is probably the major cause. The selective pressure of the antibiotic intake causes modifications in the genetic pattern of *H. pylori* that stays stable generation after generation [63]. Moreover, only a few antimicrobials are effective against this microorganism. Amoxicillin, clarithromycin, metronidazole, levofloxacin, rifabutin and tetracycline are used in triple or quadruple therapies with or without a bismuth component [1]. In particular, resistance to clarithromycin is an important predictor of treatment failure for the common eradication regimens [64]. Standard AST can be used to prescribe appropriate therapies but is currently recommended only after the second therapeutic failure [1]. *H. pylori* is a “fastidious” microorganism, with slow bacterial growth and particular nutritional requirements. For these reasons, despite new methods being developed, AST based on *H. pylori* isolation/culture remains technically challenging and time-consuming [2,65]. These factors emphasize the need for more rapid and cost-effective molecular methods that can enable a reliable prediction of the phenotypic antimicrobial resistance. The advent of molecular biology techniques has enabled the identification of molecular mechanisms underlying the observed phenotypic resistance to antibiotics in *H. pylori*. Since 1995, numerous molecular techniques have been developed. PCR-restriction fragment length polymorphism (RFLP), PCR oligonucleotide ligation assay (OLA), DNA enzyme immunoassay (DEIA), PCR line probe assay (LiPA), PCR preferential homoduplex formation assay, fluorescent in situ hybridisation and real-time PCR techniques were initially set up for the study of mutations conferring resistance to clarithromycin (whose mechanism of action was well understood), then used for other antibiotics [23,66]. Conventional methods focused on specific mutations in a small region of the target gene, and a priori knowledge of the mutations was necessary in order to use specific primers and probes, so the discovery of novel, rare or complex resistance mechanisms was limited [56].

From the early 21st century, the arising of NGS technologies has enabled a comprehensive, cost-effective, and fast tool (turnaround time from 24 to 72 h) for disease surveillance, drug resistance prediction, and evolutionary analysis of infectious diseases [67,68]. NGS includes II and III generation sequencing methods. Pyrosequencing (ROCHE 454, F. Hoffmann–La Roche Ltd., SW), the “Ion-Proton” method (Thermo Fisher Scientific Inc., MA, USA), Illumina dye sequencing with fluorophore-marked nucleotides (Illumina Inc. CA, USA) and the “DNA nanoball” method (BGI group, CH) are some of the most successful II generation techniques. PacBio real time (Pacific Bioscience Inc. CA, USA) and Oxford Nanopore (Oxford Nanopore Technologies Ltd., UK) are III generation “direct” sequencing methods, not requiring an amplification step. Currently, Illumina short reads and PacBio sequencing are the most used for bacterial genomes. The NGS of the whole genome is a powerful tool for AR prediction. WGS is a rapid method to obtain a complete view of bacterial genotypes including eventual AR-related genetic determinants. WGS-based methods are particularly useful for tracking novel, rare or complex genotypes encoding AR in clinical isolates [69], moreover, clinical relevance of new mutations can easily be assessed by retrospective analysis of WGS data [10], in fact due to the high plasticity of bacterial genome, new AR determinants emerge continuously. The application of WGS is an attractive option for genotyping the AR of *H. pylori* strains in diagnostic microbiology laboratories, especially in areas with high rates of AR, but needs to be fully understood and standardized. A major limitation of WGS is that when performed directly to gastric biopsies, results can be hampered by low bacterial DNA content and high human DNA background [5,8,70]. Moreover, not always there is a strict correlation between genotypic and phenotypic resistance. Last but not least, the relative contribution of each mutation to the MIC is not always clear; but knowing the level of antibiotic-resistance is a key factor in the clinical practice [71]. On the other hand, standard AST detect phenotypical resistance and methods are highly standardised (i.e., by EUCAST, CLSI), but they require an isolation step, biochemical tests to confirm the bacterium identity (urease, catalase and oxidase positivity for *H. pylori*); the required bacterial concentration is high (10^9^ CFU/mL *ca*.) and is not easy to obtain for a “fastidious” microorganism. Some methods can be susceptible to an inter-observer variability and in general, they fail to detect hetero-resistance status [2].

Overall, it can be stated that genotypic prediction of resistance to clarithromycin, levofloxacin and amoxicillin is accurate. For clarithromycin, the analysis of point mutations in position 2142/2143 is sufficient to monitor resistance rates in clinical practice, although other determinants are linked to the resistant status [12,15,25]. Mutations in *gyrA* gene QRDR are predictive of LEVO resistance [7], but the best results are given by the analysis of both *gyrA* and *gyrB* QRDR [5]. For AM, mutations in *pbp1A* are relevant [5], but the best genotype-phenotype correlation is obtained analysing also *hofH*, *hefC* and *hopC* genes [44].

On the other hand, for other antimicrobials, such as metronidazole, rifabutin and tetracycline, the genotype does not always match the phenotype. The causes of this lack of correlation are manifold. The prediction of metronidazole resistance based on genotypic information still remains challenging because nitroimidazoles mechanism of action is complex. All genes hypothetically related to MZ resistance encode putative MZ-reducing enzymes, DNA repair proteins, proteins regulating cell responses to oxidative stress, and outer membrane proteins [24]. In addition, phenotypic AST in vitro can overestimate MZ resistance in *H. pylori*, due to the microaerobic environment.

The main problem with tetracycline and rifabutin is the low resistance rate in *H. pylori* (globally, TC 10% and RIFA 1.5%) [43,46], this makes it difficult to obtain an accurate analysis of the genotype-phenotype relationship. Regarding TC, approximately half of the resistant isolates shows a functional tetracycline-ribosome binding site, so is highly probable that resistance results from the cumulative effects of multiple factors, not all are detectable with a strictly genetic analysis. Tetracycline resistance can be also acquired by decreased membrane permeability, and this is often coupled with additional resistance mechanisms related to cross-resistance with other drugs, (i.e., active drug efflux in *H. pylori* AM-resistant strains was related to TC cross-resistance) [72].

Regarding rifabutin, the low resistance rates, coupled with mutations in the *rpoB* gene conferring cross-resistance to other rifamycins, makes it difficult to find a strong link between genotypic and phenotypic resistance. At present, the best methodology to identify MZ, TC and RIFA resistance is based on microbiological testing.

## 4. Materials and Methods

In order to provide an overview on original research studies that focused on the characterization of *H. pylori* resistance mechanisms by whole genome NGS, a PubMed literature research was conducted. Inclusion criteria: (1) Original research manuscripts published in the last 10 years; (2) Characterization of clinical human *H. pylori* isolates; (3) Use of second and/or third generation sequencing technologies; (4) genotypic prediction of resistance to at least one of these antibiotics: CLA, MZ, LEVO, RIFA, TC. Exclusion criteria: (1) Reviews, case reports, comments, letters; (2) Characterization of non-human *H. pylori* isolates; (3) Original research manuscripts that that did not use second or third generation sequencing technology (4) text not written in English or Italian (5) studies regarding only alternative antibiotics. The quest of the terms of “*Helicobacter pylori* AND next generation sequencing AND resistance OR *Helicobacter pylori* AND whole genome sequencing AND resistance” conducted to 31 results, and the inclusion criteria were respected in 17 cases (Table 1). The literature search yielded basic research studies investigating the fundamental mechanism conferring *H. pylori* resistance vs the most used antibiotics.

## 5. Conclusions

Using genomics to investigate antibiotic resistance in bacteria of public health importance is a growing area of research; the combination of whole genome sequencing and traditional phenotypic resistance data can produce powerful results to discover new resistance mechanisms. WGS could resolve complex variants of mutations that would be challenging to detect with classical PCR-based methods, such as deletions, large insertions ending by a stop codon, and no-stop mutational changes. When the resistance is due to complex mechanisms, traditional methods, in fact, can give “false-susceptibility” results. On the other hand, this field of research has to be intended as a “work in progress”, protocols need to be standardized and the relative contribution of each mutation to the MIC as well as the effect of multiple mutations and shape transformation remains to be elucidated.

## Figures and Tables

**Table 1:** Papers considered in the literature review.

Reference	Aims	Samples	Methods	Main Finding
**(1) Tshibangu-Kabamba et al. 2020** **[5]** **(Democratic Republic of Congo)**	To explore the feasibility of genomic NGS-based approaches for tracking resistance to AM, CLA, MZ and LEVO *H. pylori* clinical isolates.	Dna extracted from 109 *H. pylori* clinical strains isolated from antral biopsies.	**AST:** agar dilution. **Sequencing:** Illumina Miseq and Hiseq platform. Pacific Biosciences (PacBio). WGS. *H. pylori* strain 26695 reference genome. All nucleotide sequences analyzed in this study were deposited in the DNA Data Bank of Japan under accession number ID: LC537338-LC537442.	WGS had a high performance in predicting phenotypic resistance to AM, CLA and LEVO. AM: based on the full-length *pbp1A* gene, mutations SAIK368_371,SKN402_404, KTG555_557, SNN559_561 in PBP-motifs and A474, T558, T593, G595 at C-terminus codons (Cohen’s Kappa = 0.842%; *p* < 0.0001). CLA: based on domain V of the 23SrRNA gene, alleles at codon-positions 2142 to 2144 (Cohen’s Kappa = 0.91; *p* < 0.0001). LEVO: based on QRDRs of the *gyrA* and *gyrB* genes (Cohen’s Kappa = 0.828; *p* < 0.0001). The prediction of MZ-R, based on full-length *rdxA* gene (functional mutations) was moderate (Youden’s index 0.64 and Cohen’s Kappa 0.30).
**(2) Tuan V.P. et al. 2019** **[56]** **(Cambodia)**	To evaluate the primary resistance of *H. pylori* to CLA, MZ, LEVO, AM and TC through NGS, and to evaluate its potential to discover genetic resistance determinants.	Dna extracted from 53 *H. pylori* clinical strains isolated from antral biopsies (naïve patients).	**AST:** agar dilution. **Sequencing:** Illumina Miseq platform. WGS. *H. pylori* strain 26695 reference genome. All genomes sequenced in this study were deposited at National Center for Biotechnology Information (NCBI) under BioProject ID PRJNA547954.	Genetic determinants were found to be significantly associated whit resistance. CLA: A2142G (*p* = 0.01) and A2143G (*p* = 0.000001) in 23SrRNA. LEVO: mutations at codon positions N87K (*p* = 0.005) and D91Y/N/G (*p* = 0.00004) in *gyrA*. AM: codons E406K (*p* = 0.005), P473L (*p* = 0.0004), and T593A/G/K (*p* = 0.005) in pbp1. Cohen’s Kappa for CLA, LEVO, AM were respectively: 0.89, 0.73, 0.73. No determinants were genetically linked to MZ (*rdxA*) or TC (16SrRNA) resistance.
**(3) Yusibova et al. 2020** **[10]** **(Denmark)**	To develop and evaluate the free CRHP Finder webtool for the detection of the most common mutations related to CLA- resistance from WGS data.	The free webtool CRHP Finder was created using 137 raw sequencing Datasets. The current study included an additional analysis of phenotypic and genotypic data from 23 *H. pylori* strains isolated from antral biopsies.	**AST:** E-test^®^. **Sequencing:** Illumina, FASTQ dataset. Sequencing data from the study are available in the ENA database with project number PRJEB37266.	CRHP Finder correctly detected all mutations reported in the previously characterized strain collection of 137 *H. pylori* strains.
**(4) Saranathan et al. 2020** **[7]** **(USA)**	To compare WGS to phenotypic testing using *H. pylori* isolates from a culture collection. Tested antibiotics: CLA, MZ, LEVO, AM, TC. Phylogenetic analysis to determine the strains lineage.	Dna extracted from 42 *H. pylori* clinical strains isolated from antral biopsies (paediatric and adult).	**AST:** E-test^®^. **Sequencing:** Illumina Miseq platform. WGS. *H. pylori* strain 26695 reference genome. All sequenced genomes were submitted to NCBI BioProject database under BioProject accession number PRJNA566177.	Phenotypic resistance correlated with the presence of alleles of 23S rRNA (A2142G/A2143G) for CLA (Cohen’s Kappa= 0.84) and *gyrA* (N87I/N87K/D91Y/D91N/D91G/D99) for LEVO (Cohen’s Kappa = 0.90). Phenotypic resistance to AM was observed only in three isolates, carriyng mutations in *pbp1*, *pbp2*, and/or *pbp3* (within coding regions near known PBP motifs). All isolates were phenotypically susceptible to TC. Potential genetic determinants of rifampin (any mutation in *rpoB,* Cohen’s Kappa = 0.46), and MZ (truncation in *rdxA* or an R16H mutation, Cohen’s Kappa = 0.76) resistance need further investigation.
**(5) Egli et al. 2020** **[8]** **(Switzerland)**	To compare the diagnostic performance of 23SrDNA qPCR and *gyrA* qPCR followed by Sanger sequencing with a IVD-marked hybridization probe assay and with WGS, for the detection of LEVO-R in *H. pylori* isolates..	DNA extracted from 142 gastric biopsies and 76 *H. pylori* clinical isolates.	**AST:** E-test^®^. **Sequencing:** Sanger sequencing by Microsynth. Illumina Miseq platform. WGS. *H. pylori* strain 26695 reference genome. The datasets generated for this study can be found in NCBI GenBank, NCBI Accession No. MW057345-51.	Application of qPCR (23SrDNA and *gyrA*) and Sanger sequencing (*gyrA*) on biopsies can overcome disadvantages of cultures and should be applied for the detection of *H. pylori* resistance to CLA and LEVO in gastric biopsies. WGS correctly detect all 48 LEVO-S strains and 27/28 Levo-R strains (mutations in codon position N87, D91). WGS correctly identified all 27 CLA-S and 49 CLA-R isolates (A2142G/C and A2143G).
**(6) Chen et al. 2018** **[15]** **(China)**	To characterize the multidrug efflux transporter gene variants in the 23SrRNA mutations in clinical isolated *H. pylori* strains.	DNA extracted from 12 *H. pylori* clinical isolates.	**AST:** E-test^®^ and agar dilution. **Sequencing:** Illumina HiSeq 2500 platform. WGS. Reference *H. pylori* strain 26695. Sequencing data are available from the corresponding author on reasonable request.	NGS of clinical isolated *H. pylori* is a useful method for identifying genome variations. Analysis of multidrug efflux transporter gene mutation indicated that membrane proteins of RND family possibly play an indispensable role in resistance to CLA. Total SNVs of multidrug efflux transporter gene and the SNVs of HP0605 (TolC homologue) were significantly different (*p* ≤ 0.05) between CLA-R and CLA-S strains. There was no significant difference in SNVs of RND or MFS (major facilitator superfamily) between CLA-R and CLA-S.
**(7) Iwamoto et al. 2014** **[12]** **(Japan)**	To identify single nucleotide variants of multi-drug resistant efflux pump genes in the CLA-resistant phenotype.	DNA extracted from 12 *H. pylori* clinical isolates and ATCC26695 reference strain.	**AST:** agar dilution. **Sequencing:** Illumina MiSeq platform. WGS. *H. pylori* strain 26695 reference genome. All sequence reads were deposited in the DNA Data Bank of Japan Sequence Read Archive under accession number DRA001250.	RND efflux pump systems are involved in CLA resistance status. WGS sequencing has yielded novel understanding of genotype-phenotype relationships. There were significant differences in SNVs of the hp0605-hp0607 cluster between susceptible and resistant strains (*p* < 0.05), but not in the other clusters.
**(9) Lauener et al. 2019** **[25]** **Switzerland**	To compare phenotypic AST results with the predictions based on the presence of genetic determinants identified in the *H. pylori* genome using WGS. Phenotypic resistance to CLA, MZ, TC, LEVO and rifampicin was determined.	DNA extracted from 140 *H. pylori* strains from the bacterial strain collection of the Institute of Medical Microbiology, University of Zurich.	**AST:** E-Test^®^ **Sequencing:** Illumina MiSeq platform. WGS. *H. pylori* strain 26695 reference genome. Whole genome sequences of the *H. pylori* strains analyzed in this study are available on NCBI (accession numbers reported in Supplementary materials of the quoted paper).	There was a high congruence of >99% between phenotypic AST results for CLA, LEVO and rifampicin and SNPs in 23SrRNA, *gyrA* and *rpoB* genes. CLA: the presence of mutations A2142C/G and A2143G were significantly related to CLA-R (*p* < 0.001). LEVO: specific amino acid exchanges at codon 87 (N87I, N87K and N87Y) and 91 (D91N and D91Y) of the *gyrA* gene were highly predictive of LEVO –R (*p* < 0.001). Only four *H. pylori* isolates were resistant to rifampicin, they had either an H540N (*n* = 2) or an L525P (*n* = 2) amino acid exchange. MZ: Some frameshift mutations (at codon positions, 105, 149 and 192 in *frxA* and 18, 38 and 112 in *rdxA*) were only detected in MZ-R strains, other frameshift mutations/SNP in *frxA* and *rdxA* occurred both in MZ-R and MZ-S isolates, it was not possible to infer a resistance phenotype for MZ based on the occurrence of distinct SNPs in *frxA* and *rdxA.* All isolates analysed in this study were susceptible to TC, in accordance with the absence of nucleotide substitutions in the 16SrRNA gene.
**(10) Azzaya et al. 2020** **[43]** **(Mongolia)**	To investigate the prevalence of antibiotic resistance and its underlying genetic in Mongolia. Antibiotics: AM, CLA, MZ, LEVO, and minocycline.	Dna extracted from 74 *H. pylori* clinical strains.	**AST:** agar dilution. **Sequencing:** Illumina MiSeq platform. WGS. *H. pylori* strain 26695 reference genome. All nucleotide sequences analyzed in this study were deposited in the DNA Data Bank of Japan under accession number ID: LC567134-LC567141, LC567329-LC567379, LC568549-LC568586.	The rate of antibiotic resistance of *H. pylori* infections was high, particularly to AM, CLA, MZ, and LEVO. Novel mutations in the *pbp1A* as well as in the 23SrRNA gene were detected. N562Y (adjacent to motif KTG555-7) was significantly associated with AM resistance (*p* = 0.00001). A2143G was significantly related to CLA resistance (*p* = 1.99 × 10^−^^11^). Amino acid substitutions at positions N87K (*p* = 0.00009), D91G (*p* = 5.1 × 10^−^^9^), D91N (*p* = 0.00002) and D91Y (*p* = 0.001) were significantly associated with LEVO resistance. NGS provided a powerful tool for determining antibiotic-resistance for CLA, AM, LEVO.
**(11) Qumar et al. 2020** **[31]** **(Bangladesh)**	To investigate the prevalence of distinct genotypes of *cagA, vacA*, and *babA/B* and other virulence factors, together with genotype-based antibiotic resistance profiles in prevailing Bangladeshi *H. pylori* lineages. To find the association with clinical outcome.	Dna extracted from 20 *H. pylori* isolates randomly extracted from a cohort of 174 (125 adult and 49 children) symptomatic or asymptomatic patients.	**AST:** genotype-based, in silico. **Sequencing:** Illumina MiSeq platform. WGS. *H. pylori* strain 26695 reference genome.	The study showed a high occurrence of MZ- and LEVO-R *H. pylori* strains in Bangladesh. Strains with similar antibiotic resistance pattern could be separated into two major population with distinct *cagA* and *vacA* genotypes (HpAsia2 and HpEurope). 50% of MZ resistant strains were expected to express non-functional or altered RdxA and/or FrxA proteins. A N-terminal extension of GyrA by five amino acid residues (QDNSV) and amino acid exchanges in QRDR occurred solely in LEVO-R *H. pylori.*
**(12) Domanovich-Asor et al. 2021** **[26]** **(Israel)**	To conduct a comprehensive genotypic-phenotypic comparison among a set of Israeli *H. pylori* isolates. Antibiotics considered: AM, CLA, LEVO, MZ, and rifampicin.	Dna extracted from 48 *H. pylori* clinical isolates.	**AST:** E-test^®^. **Sequencing:** Illumina iSeq 100 or Miseq platforms. WGS. *H. pylori* strain 26695 reference genome. Sequence data deposited to BioProject ID PRJEB37854.	In this study novel point mutations were discovered among phenotypically-resistant isolates: G94E at the *pbp1A* gene; C2173T and G2212A at the 23SrRNA gene; T239M at the *gyrA* gene; G122R at the *rdxA* gene; A70T and A138V at the *frxA* gene. Overall results demonstrated a complicated relationship between genotype and phenotype, this supports the need for future research.
**(13) Miftahussurur et al. 2019 [50] (Bangladesh and Nepal)**	To evaluate the susceptibility and genetic mutations of 5 alternative antibiotics in the treatment of *H. pylori,* against isolates from Nepal and Bangladesh. Antibiotics considered: furazolidone, rifabutin, rifaximin, sitafloxacin, garenoxacin.	DNA extracted from 98 *H. pylori* clinical isolates (56 from Bangladesh, 42 from Nepal).	**AST:** agar dilution. **Sequencing** Illumina MiSeq platform *H. pylori* strain 26695 reference genome. These sequence data have been submitted to the DDBJ databases under accession number: LC425712-LC425829.	No resistance to furazolidone or rifabutin and a high susceptibility of sitafloxacin were observed. Resistance to rifaximin and garenoxacin was high. Mutations of *gyrA* could play a significant role in garenoxacin resistance, and double mutations of A87 and D91 were associated with sitafloxacin resistance. Analysis of the *rpoB* gene demonstrated well-known mutations, such as V657I, and several novel mutations, including I2619V, V2592L, T2537A, and F2538L.
**(14) Chua et al. 2019 [27] (Australia)**	To detect additional, uncharacterized, mechanisms of MZ resistance in *H. pylori*.	DNA extracted from 121 *H. pylori* clinical strains.	**AST:** E-test^®^. **Sequencing** Illumina MiSeq platform. WGS. *H. pylori* strain 26695 reference genome. All draft genomes have been deposited at DDBJ/ENA/GenBank and all sequencing data generated in this study have been submitted to Sequence Read Archives (SRA) database. Accession numbers are listed in Supplementary Table 1 of the quoted paper.	Resistance due to RdxA truncation was identified in 34% of MZ-R strains. The distribution of RdxA inactivation in MZ-R strains was statistically significant (*p* < 0.001). The distribution of FrxA inactivation was significantly greater in the MZ-R strains (*p* = 0.008), indicating that FrxA truncation might play a role in MZ-resistance. Amino acid substitution of Arg-16 in RdxA was statistically significant (*p* = 0.038). Four protein clusters were found to harbour a variable site in which the distribution of amino acid variants was significantly greater among the MZ-R strains; these substitutions included R16H/C in RdxA (*p* = 0.038), D85N in the inner-membrane protein RclC (*p* = 0.021), V265I in a biotin carboxylase protein (*p* = 0.047) and A51V/T in HP0918 (*p* = 0.006). These results help to explain the varying levels of MZ resistance in different *H. pylori* strains.
**(15) Qureshi et al. 2014** **[44]** **California**	To track in vitro the evolution of AM resistance in *H. pylori,* and identify a variety of genes, which can contribute to this resistance.	DNA extracted from reference strain ATCC26695 at 5 different times (IS1, 2, 3, 4, 5) during induction of AM-resistance.	**AST** broth microdilution. **Sequencing:** Illumina platfrom. WGS. *H. pylori* strain 26695 reference genome.	MIC observed is isolate 5 was 64x higher than the parental strain’s MIC value. The majority of high-level AM-resistance can be explained by the combined effects of amino acid changes occurring in PBP1, HopC, HefC, HofH, and possibly PBP2. Mutations contributing to the increase in MIC were: P372S in PBP1 (IS1); R302H in HopC (IS2), L378F in HefC (IS3); T438M in PBP1 (IS4). The ulterior increase in MIC observed in IS5 is caused by a combination of both PBP2 and HofH mutations. This study demonstrated the significance of the HofH mutation at G228W. It remains possible that additional mutations can also contribute to amoxicillin resistance.
**(16) Miftahussurur et al. 2019** **[51]** **Japan**	To determine the resistance rates of five alternative antibiotics for *H. pylori* (rifaximin, rifabutin, furazolidone, garenoxacin, and sitafloxacin) and to assess mutations associated with antibiotic resistance using NGS.	Dna extracted from 106 *H. pylori* clinical isolates derived from 1039 adult dyspeptic patients.	**AST.** agar dilution. **Sequencing:** Illumina MiSeq platform. WGS. *H. pylori* strain 26695 reference genome. The nucleotide sequences were deposited in the DDBJ under accession numbers LC420463–LC420466 (*gyrA* and *gyrB*), and LC420467– LC420511 (*rpoB*).	DNA sequence analysis of *rpoB* from all rifaximin-sensitive strains revealed intact reading frames. Among all 2890 codons of *rpoB*, 1010 had silent mutations. The majority of the rifaximin-resistant strains (97.5%) contained missense mutations. The predominant point mutations of *rpoB* were I837V (20%), A2414T/V (20%), Q2079K (17.5%), and K2068R (17.5%). There was an association between the *vacA* genotype of *H. pylori* with rifaximin resistance (*p* = 0.048). These mutations did not confer resistance to rifabutin.
**(17) Binh et al. 2014** **[24]** **Vietnam**	To identify novel mutations leading to MZ resistance, by inducing MZ resistance in vitro.	DNA extracted from a MZ-resistant strain (derived from MZ-susceptible *H. pylori* strain 26695 by exposure to low concentrations of the drug).	**AST** E-test^®^ and agar dilution. **Sequencing** Illumina HiSeq 2000 platform. WGS. *H. pylori* strain 26695 reference genome. Genome sequences of wild-type 26695-1 and MZ-resistant strain 26695-1MET were deposited at GenBank under accession no. CP010435 and CP010436, respectively.	Mutations in the *rdxA* gene are mainly associated with MZ-R, whereas mutations in the *frxA* gene enhance *H. pylori* resistance exclusively in the presence of *rdxA* mutations. Using natural transformation, C46T, G238A and G352A in the *rdxA* gene led to a moderate MZ-R (MIC 48 mg/L). Mutation G3A led to a low MZ-R (MIC 16 mg/L), but when coupled with indel -571TA in *frxA,* the MIC increased to 48 mg/L. The ribosomal gene *rpsU* (mutation G37T) may be an additional candidate associated with MZ resistance.

## Data Availability

Data available in a publicly accessible repository. The data presented in this study are openly available in the Pub Med database.

## Abbreviations

**A** = Adenine; **C** = Cytosine; **G** = Guanine; and **T** = Thymine; **A** = Alanine; **C** = Cysteine; **D** = Aspartic acid; **E** = Glutamic acid; **F** = Phenylalanine; **G** = Glycine; **H** = Histidine; **I** = Isoleucine; **K** = Lysine; **L** = Leucine; **M** = Methionine; **N** = Asparagine; **P** = Proline; **Q** = Glutamine; **R** = Arginine; **S** = Serine; **T** = Threonine; **V** = Valine; **W** = Tryptophan; and **Y** = Tyrosine.
